# Temperature monitoring: An often neglected but essential standard monitor

**DOI:** 10.4103/0019-5049.76580

**Published:** 2011

**Authors:** Indira Malik

**Affiliations:** Department of Anaesthesiolgy and Critical Care, Govind Ballabh Pant Hospital, New Delhi, India

Sir,

I read with interest the recent case report by Kalra.[1] titled “Role of amino acid infusion in delayed recovery from neuromuscular blockers”. I admit that it was difficult to establish a differential diagnosis in this case regarding the cause of excessive sedation and unresponsiveness seen in the post operative period. The authors have been resourceful indeed in using amino acid infusion to correct hypothermia, which is a very novel approach.

However, I feel that this particular situation could have been avoided had there been a continuous monitoring of body temperature intraoperatively. Because this is included among standard monitors,[2] one must always ensure that a temperature probe be used for all procedures being performed under any type of anaesthesia. Elderly and frail patients, as this patient definitely was, are extremely prone to develop hypothermia even during short duration procedures. So it was all the more important to monitor her temperature. Most of our current monitors have either a nasopharyngeal or surface temperature probe and, so now it is not at all cumbersome to monitor the thermal status of patients.

Another point that is worthy of discussion is that the dose of fentanyl could have been reduced as we all know that it is highly extracted by the liver and, therefore, its clearance depends on the hepatic blood flow, which is positively reduced in old age. It is preferable to reduce the dosage of fentanyl by 50% in these patients.[3] This would reduce the incidence of bradycardia and respiratory depression seen in the post anaesthesia care unit.
